# Protein Synthesis by Day 16 Bovine Conceptuses during the Time of Maternal Recognition of Pregnancy

**DOI:** 10.3390/ijms21082870

**Published:** 2020-04-20

**Authors:** Irene Malo Estepa, Haidee Tinning, Elton Jóse Rosas Vasconcelos, Beatriz Fernandez-Fuertes, José María Sánchez, Gregory W. Burns, Thomas E. Spencer, Pat Lonergan, Niamh Forde

**Affiliations:** 1 Discovery and Translational Sciences Department, Leeds Institute of Cardiovascular and Metabolic Medicine, Faculty of Medicine and Health, University of Leeds, West Yorkshire LS2 9JT, UK; I.MaloEstepa@leeds.ac.uk (I.M.E.); bs13hghs@leeds.ac.uk (H.T.); 2 Leeds Omics Virtual Research Institute, University of Leeds, West Yorkshire LS2 9JT, UK; E.Vasconcelos@leeds.ac.uk; 3 Department of Biology, Faculty of Sciences, Institute of Food and Agricultural Technology, University of Girona, 17003 Girona, Spain; bffuertes@gmail.com; 4 School of Agriculture and Food Science, University College Dublin, Belfield, Dublin 4, Ireland; jovenpadawan5@hotmail.com (J.M.S.); pat.lonergan@ucd.ie (P.L.); 5 Division of Animal Sciences, University of Missouri, Columbia, MO 65211, USA; gbvd6@mail.missouri.edu (G.W.B.); spencerte@missouri.edu (T.E.S.)

**Keywords:** endometrium, SILAC, pregnancy recognition, conceptus, extracellular vesicles, proteomics, bovine

## Abstract

Interferon Tau (IFNT), the conceptus-derived pregnancy recognition signal in cattle, significantly modifies the transcriptome of the endometrium. However, the endometrium also responds to IFNT-independent conceptus-derived products. The aim of this study was to determine what proteins are produced by the bovine conceptus that may facilitate the pregnancy recognition process in cattle. We analysed by mass spectrometry the proteins present in conceptus-conditioned media (CCM) after 6 h culture of Day 16 bovine conceptuses (*n* = 8) in SILAC media (arginine- and lysine-depleted media supplemented with heavy isotopes) and the protein content of extracellular vesicles (EVs) isolated from uterine luminal fluid (ULF) of Day 16 pregnant (*n* = 7) and cyclic (*n* = 6) cross-bred heifers on day 16. In total, 11,122 proteins were identified in the CCM. Of these, 5.95% (662) had peptides with heavy labelled amino acids, i.e., de novo synthesised by the conceptuses. None of these proteins were detected in the EVs isolated from ULF. Pregnancy-associated glycoprotein 11, Trophoblast Kunitz domain protein 1 and DExD-Box Helicase 39A were de novo produced and present in the CCM from all conceptuses and in previously published CCM data following 6 and 24 h. A total of 463 proteins were present in the CCM from all the conceptuses in the present study, and after 6 and 24 h culture in a previous study, while expression of their transcripts was not detected in endometrium indicating that they are likely conceptus-derived. Of the proteins present in the EVs, 67 were uniquely identified in ULF from pregnant heifers; 35 of these had been previously reported in CCM from Day 16 conceptuses. This study has narrowed a set of conceptus-derived proteins that may be involved in EV-mediated IFNT-independent embryo–maternal communication during pregnancy recognition in cattle.

## 1. Introduction

Understanding the molecular communication that occurs between the developing conceptus (embryo and associated extraembryonic membranes) and the maternal endometrium is key to enhancing our understanding of early pregnancy loss in cattle. During early pregnancy, the embryo induces very localised changes at the transcriptional level in the oviduct [1,2] and in vitro coculture of embryos with oviductal cells accelerates blastocyst development and changes the epigenetic status of the embryos [2,3]. This indicates that interaction with the female reproductive tract, while not strictly necessary for early embryo development, enhances embryo quality [4]. More recently, local effects of the embryo on the endometrium at the blastocyst stage have been demonstrated [5,6,7]. Following hatching of the blastocyst from the zona pellucida, there begins a sequential modification to the transcriptome of the endometrium as early as Day 13 [8,9], which is amplified during the time of pregnancy recognition (Day 16) [10,11,12] This response is further propagated into extrauterine tissues, including the corpus luteum (CL) and peripheral blood [13,14,15,16]. These interactions are predominantly mediated by the pregnancy recognition signal Interferon Tau (IFNT), a type-I interferon produced by the trophectoderm cells which is required to inhibit luteolysis and maintain the CL. In addition to IFNT, which significantly modifies the transcriptome of the endometrium [17,18], other proteins are produced by the conceptus [19,20] coordinated with increased production of IFNT. Moreover, the response of the endometrium to the conceptus as a whole is greater than its response to prolonged exposure to a type I interferon for 3 days in vivo [12,18]. 

In addition to traditional receptor–ligand binding signaling between the endometrium and the conceptus, there is evidence for a role of extracellular vesicle (EV)-mediated communication between the conceptus and the endometrium in sheep [21,22] and cattle [23] during early pregnancy. The size, concentration, and content of EVs released by the embryo can be affected by environmental factors, such as oxidative stress, in an embryo sex-dependent manner [24]. This type of communication may also explain, in part, why a large proportion of uterine lumen fluid (ULF) proteins are not classically secreted in nature, i.e., not classed as ligands or extracellular proteins [19]. 

Studying the proteins (other than IFNT) that may mediate conceptus–maternal interaction is challenging for a number of reasons. Firstly, proteins that are present in the ULF around the time of pregnancy recognition may be derived from the conceptus itself or may be derived from the endometrium in response to the presence of the conceptus, or a combination of both [25]. Secondly, attempts to grow embryos past the blastocyst stage and artificially induce elongation in vitro have not been successful [26,27] and therefore in vivo models are essential. We have previously characterized the proteins produced by the conceptus following culture in vitro for 6 and 24 h; however, which of these proteins are de novo synthesized as a response to the in vitro environment or facilitate conceptus–maternal interactions is not known [19]. 

One approach to the identification of de novo synthesized proteins is to use a technique known as stable isotope labelling by amino acids in cell culture (SILAC). This approach consists of heavy or light isotope-labelled amino acids that are added to cell culture media depleted of these specific amino acids. These heavy forms of amino acids then get incorporated during protein synthesis in vitro, i.e., they are de novo synthesized [28]. Because of the difference in mass of the peptides produced bearing the heavy forms of these amino acids, we can differentiate these via mass spectrometry. This allows the identification of de novo produced proteins, i.e., those containing the heavy isotope forms of amino acids in their peptide fragments [28]. This widely used technique has proven useful in the study of de novo produced proteins in vitro [29,30,31,32] and could bring novel insights into conceptus-derived proteins involved in pregnancy recognition. 

The aim of the present study was to use this novel SILAC-based approach to identify de novo produced proteins by the Day 16 bovine conceptus and to determine if any of these proteins are present in the EVs derived from the ULF of pregnant heifers, which would support a role in conceptus–maternal interactions during pregnancy recognition. 

## 2. Results

### 2.1. Experiment 1

#### 2.1.1. Proteins Present in Conceptus-Conditioned Media following 6 h Culture of Day 16 Conceptuses in Vitro

Proteomic analysis of CCM identified 11,122 different proteins present in the media recovered from one or more conceptuses (numbers and percentages are shown in Table 1) that were not detected in the contemporaneous blanks. Of those, 1576 proteins (14.2%) were identified in media from all eight conceptuses, 2757 (24.8%) were present in media recovered from six or more conceptuses (*n* ≥ 6), and 3997 (36.0%) proteins identified in media recovered from at least four of the conceptuses (*n* ≥ 4; Supplementary Table S1). Six different isoforms of IFNT were detected in the CCM from different conceptuses (Supplementary Table S1). These were IFNT 1i, IFNT 1g, IFNT 3i, IFNT 3, IFNT 4a and IFNT c1. The only heavy labelled variant was IFNT variant 1g in the CCM from conceptus “H”. The rest of CCM presented one or more light labelled variants of IFNT.

The 1576 proteins detected in CCM from all eight conceptuses were analyzed using DAVID to identify over-represented biological processes and pathways (Supplementary Table S2). The top 10 over-represented KEGG pathways of the 77 detected based on their fold enrichment were mainly related to metabolism (Table 2). Gene ontology (GO) term analysis for biological processes identified 492 over-represented terms, the details of which are provided in Supplementary Table S3.

#### 2.1.2. De novo Proteins Synthesized by Day 16 Conceptuses following Culture in Vitro

A total of 662 proteins were identified as having peptide fragments containing heavy labelled amino acids (5.95% of the total proteins, Table 1). This is indicative of being de novo produced by the conceptuses in vitro as opposed to those light labelled, which were most likely produced prior to culture of the conceptuses in SILAC media. Proteins were heavy labelled to different extents depending on the sample analyzed. No protein containing heavy labelled amino acids was detected in CCM from all eight conceptuses (Table 3). THO complex subunit 7 homolog (THOC7) and RING finger protein 207 (RNF207), containing heavy labelled amino acids in their peptide fragments, were detected in CCM from five animals, with SET domain containing 9 (SETD9) detected in CCM from four animals. Six de novo produced proteins (MER proto-oncogene tyrosine kinase (MERTK); G protein-coupled receptor class C group 5 member A (GPRC5A); DExH-box helicase 34 (DHX34); Disco interacting protein 2 homolog A (DIP2A); MAGE domain-containing protein (MAGED1); and OTU deubiquitinase 6A (OTUD6A)) were identified in media from three conceptuses, and a total of 42 proteins with heavy labelled amino acids in their peptides were de novo produced by two conceptuses (Table 1). A total of 611 proteins with heavy labelled amino acids were present in the CCM from one conceptus (Supplementary Table S1). Three proteins, MERTK, and Poly (ADP-ribose) polymerase (TNKS2) and Trophoblast Kunitz domain protein 1 (TKDP1), were detected in CCM in all eight samples; however, heavy labelled versions of these proteins were only detected in two samples. Those proteins that were identified in CCM of more than three conceptuses that contained a heavy labelled peptide fragment are presented in Table 3.

Analysis of those proteins that contained peptides with heavy labelled amino acids revealed KEGG pathway over-representation of four pathways related to secretion and steroidogenesis (Table 4). No GO terms were over-represented (Supplementary Table S4). For the 26 proteins heavy labelled to a different extent (i.e., heavy labelled in one or more CCM) and present in the media from all eight conceptuses, no specific KEGG pathways or GO terms were identified as over-represented, likely due to the short list of proteins. 

### 2.2. Experiment 2

#### 2.2.1. Specific Proteins are Unique to Extracellular Vesicles Recovered from Uterine Luminal Fluid of Pregnant Heifers on Day 16

**Figure 1:**
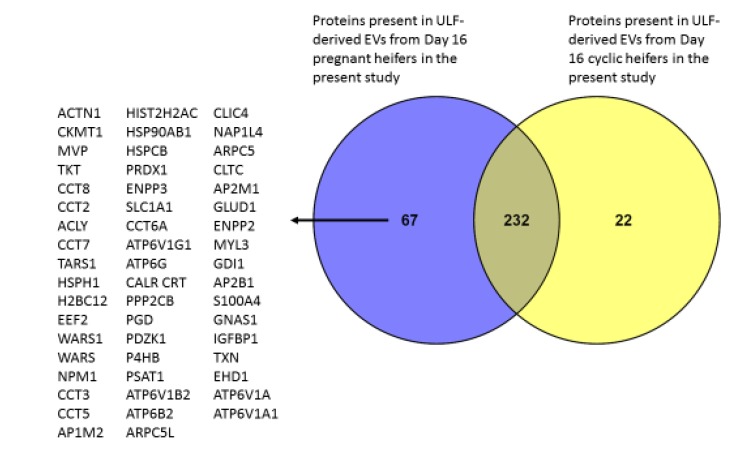
Venn diagram of the proteins present in extracellular vesicles (EVs) from the uterine luminal fluid (ULF) via nano-LC MSMS proteomics from Day 16 pregnant (*n* = 6) and cyclic (*n* = 5) heifers. Differences were identified between pregnant (Preg Day16 EVs) and cyclic (Cyc Day 16 EVs).

The analysis of EVs isolated from ULF of confirmed pregnant and cyclic heifers in Experiment 2 had a mean (± sd) diameter of 135 nm (± 15.8 nm) with a range of 111–162 nm. The average concentration of EVs was 1.13 × 10^12^ particles/mL (Table 5). In total, 324 proteins were identified with high confidence, i.e., 99% identity and with at least two spectral counts per protein (Supplementary Table S5). All data were filtered to identify those proteins present in the ULF of at least four of six heifers (for the pregnant group) and four of five heifers (in the cyclic group). A total of 301 and 256 proteins were identified in EVs recovered from the ULF of pregnant and cyclic heifers, respectively. Of these proteins, 232 were common to both groups, while 67 and 22 were unique to pregnant and cyclic ULF, respectively (Figure 1). Of the 67 proteins only identified in pregnant ULF, 35 were previously detected in CCM following short-term culture of Day 16 conceptuses, while ten of the 22 proteins detected in cyclic EVs were also detected in CCM [19].

The 67 proteins present exclusively in the EVs from ULF of pregnant heifers were submitted for KEGG pathway enrichment analysis. This revealed 11 over-represented pathways (Supplementary Table S6) involved in order by fold enrichment (Table 6) collecting duct acid secretion, synaptic vesicle cycle, endocrine and other factor-regulated calcium reabsorption, tight junction, rheumatoid arthritis, systemic lupus erythematosus, carbon metabolism, phagosome, endocytosis, protein processing in endoplasmic reticulum, and biosynthesis of antibiotics. The 10 most over-represented GO terms of 28 biological processes were mainly related to protein production and chromosomal alignment, with details provided in Supplementary Table S7.

#### 2.2.2. Comparison between Conceptus-Derived Proteins and Those Present in the EVs from ULF

**Figure 2:**
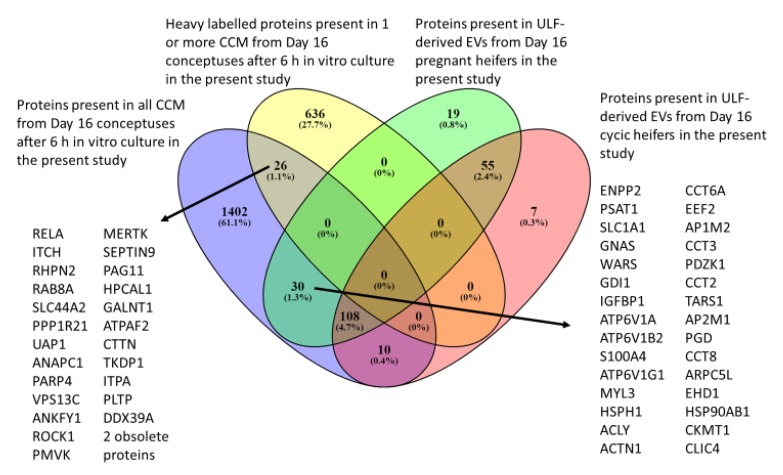
Venn diagram of the proteins present in CCM via nano-LC MSMS proteomics of SILAC-labelled media minus Arginine and Lysine supplemented with heavy forms of these amino acids (*n* = 8 CCM, *n* = 3 contemporaneous blanks) from Day 16 in vivo produced conceptuses after 6 h culture, and the proteins present in EVs from ULF via nano-LC MSMS proteomics from Day 16 pregnant and cyclic heifers.

The GI identifiers of all proteins from EVs (Experiment 2) were converted to UniProt accession IDs using the retrieve/ID mapping tool from Uniprot.org in order to allow comparison of those present in EVs recovered from ULF of cyclic and pregnant heifers on Day 16 to those present in CCM from the current study (Experiment 1; Figure 2). After Uniprot conversion, 212 and 180 proteins were retrieved as present in EVs from the ULF of pregnant and cyclic heifers, respectively. Venn diagram analysis identified 108 proteins that were common to CCM and EVs derived from ULF of both pregnant and cyclic animals. Thirty proteins were present in CCM and EVs derived from ULF from pregnant heifers. In contrast, only 10 proteins were common between CCM and EVs derived from ULF of cyclic animals (Figure 2). Heavy proteins were only present in CCM and only 26 of them were actually present in the media from all the eight conceptuses. No overlap in proteins with heavy labelled amino acids was present in the EVs from the ULF.

### 2.3. Comparison with Previous Studies

#### 2.3.1. Comparison between Conceptus-Derived Proteins and Previously Published Work on CCM

**Figure 3:**
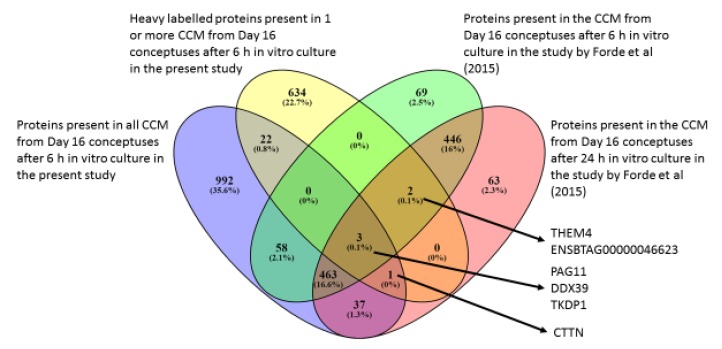
Venn diagram of the proteins present in CCM via nano-LC MSMS proteomics of SILAC-labelled media minus Arginine and Lysine supplemented with heavy forms of these amino acids (*n* = 8 CCM; *n* = 3 contemporaneous blanks) from Day 16 in vivo produced conceptuses after 6 h culture. Differences were identified between All CCM determined proteins, those incorporated with Heavy labelled amino acids and previously reported data in the literature following 6 or 24 h culture [19].

The proteins (total and heavy labelled) detected in the CCM from all eight conceptuses in the current study were compared to those detected in CCM after 6 and 24 h culture in non-SILAC media in our previous study (Figure 3) [19]. Only six heavy labelled proteins were detected in the previous study. Pregnancy associated protein 11 (PAG11), DEAD (Asp-Glu-Ala-Asp) box polypeptide 39 isoform 1 (DDX39) and TKDP1 were heavy labelled to different extents and present in the CCM from all the eight conceptuses in the present study and also in CCM after 6 and 24 h culture. Cortactin (CTTN) was present in the CCM from all conceptuses in the present study, was heavy labelled and also present in CCM after 24 h culture in the previous study. Two other proteins, Acyl-coenzyme A thioesterase (THEM4) and an uncharacterized protein (ENSBTAG00000046623), were heavy labelled to different extents in the present study and also present in CCM after 6 and 24 h culture of Day 16 conceptuses in the previous study, THEM4 was present in only seven of the eight CCM in the present study. 

A group of 463 proteins present in CCM from all the eight conceptuses in the current study did not have peptides with heavy labelled amino acids, and were also identified in CCM following 6 and 24 h culture in a previous study [19]. These 463 proteins were subjected to KEGG pathway analysis (Table 7) [33,34] and GO term analysis [35,36,37]. Thirty pathways were over-represented in this group (Supplementary Table S8). A total of 297 GO biological terms were over-represented (Supplementary Table S9), mainly related to chromosome alignment. None of the transcripts coding for these 463 proteins were detected in the endometrium in a previous study [9], indicating that they are likely of conceptus origin in ULF.

**Figure 4:**
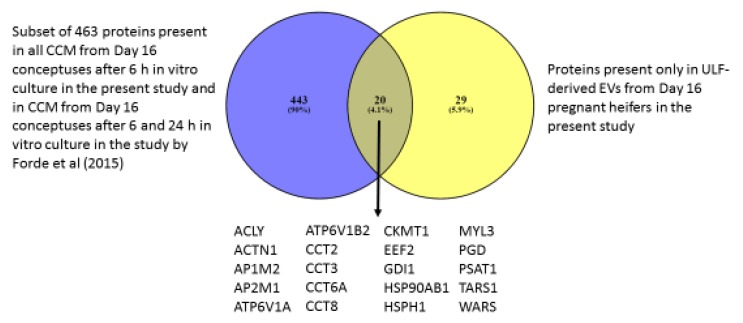
Venn diagram of the subset of 463 proteins identified via nano-LC MSMS proteomics in the CCM of Day 16 pregnant heifers after 6 h of culture and present after 6 and 24 h culture in a previous study [19] compared to those present in EVs from ULF of Day 16 pregnant heifers only.

**Figure 5:**
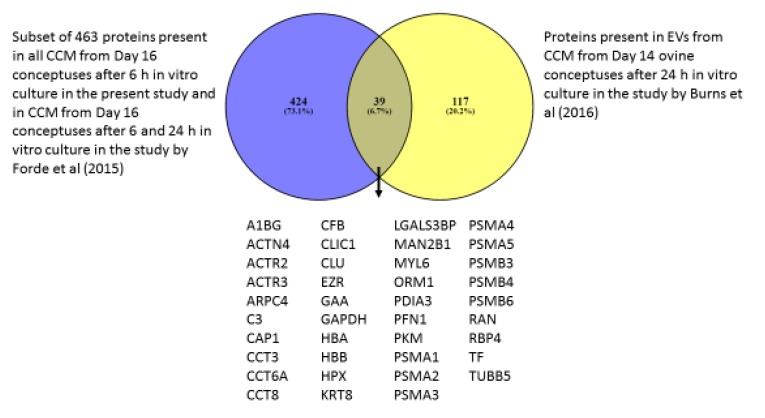
Venn diagram of the subset of 463 proteins identified via nano-LC MSMS proteomics in the CCM of Day 16 pregnant heifers after 6 h of culture and present after 6 and 24 h culture in a previous study [19] compared to those present in EVs from CCM of Day 14 pregnant ewes after 24 h in vitro culture [38].

The subset of 463 proteins present in the CCM from all the conceptuses in this study and detected following 6 and 24 h culture in a previous study [19] were compared to those only present in the EVs from ULF of Day 16 pregnant heifers (49 proteins) and there was an overlap of 20 proteins (Figure 4). These 463 proteins were also compared to the proteins detected in EVs from CCM after 24 h Day 14 ovine conceptuses in vitro culture (Figure 5). A total of 39 proteins were common between both species. 

#### 2.3.2. Comparison of EV Components to Protein Composition of ULF from Previously Published Studies

**Figure 6:**
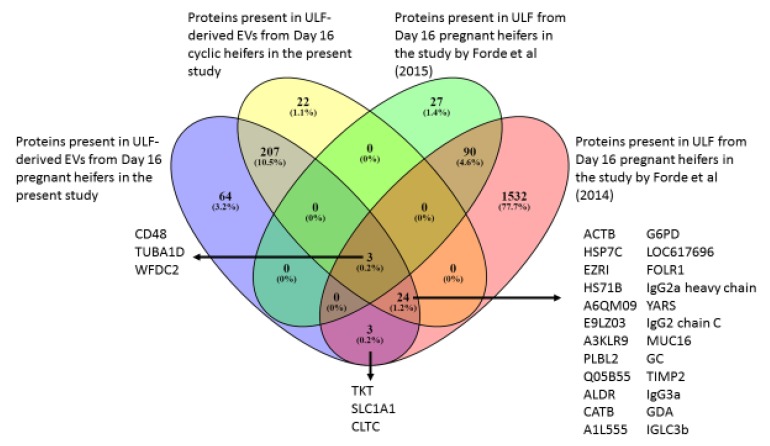
Venn diagram of the proteins present in EVs from ULF via nano-LC MSMS proteomics from Day 16 pregnant and cyclic heifers. Differences were identified between pregnant (ULF-P, *n* = 6), cyclic (ULF-C, *n* = 5) and previously reported data in clarified ULF from Day 16 pregnant heifers [19,25].

The proteins present in the EVs from ULF of pregnant (301) and cyclic (256) heifers identified in the present study were compared to those previously identified in ULF on Day 16 of pregnancy in heifers not subjected to EV isolation [19,25]. Three proteins (CD48 molecule, tubulin alpha-1D chain, TUBA1D, and WAP four-disulfide core domain 2, WFDC2) were present in all groups, independent of EV enrichment or pregnancy status. Furthermore, 24 proteins were present in EVs from pregnant and cyclic heifers, as well as in ULF from Forde, McGettigan [25]. Finally, three proteins (TKT protein, amino acid transporter, SLC1A1, and Clathrin heavy chain 1, CLTC) were present in EVs from pregnant heifers and ULF from Forde, McGettigan [25]. These data are presented in Figure 6.

## 3. Discussion

Understanding the signaling that occurs between the conceptus and the endometrium during maternal recognition of pregnancy is complex, particularly when investigating the role of conceptus-derived proteins other than IFNT. Understanding what proteins are produced and which of these are involved in EV-mediated interaction is complex due to the numerous sources of Day 16 proteins in ULF in vivo and the limitations around conceptus culture in vitro. To attempt to address this, we used a SILAC-labelled media approach to try to identify de novo produced proteins (and possibly a consequence of the in vitro environment) and those that are produced by the conceptus that may induce IFNT-independent responses in the endometrium. We also sought to determine if any of these are components of EVs present in the ULF of pregnant heifers. 

Using a SILAC approach, only a small proportion of the proteins detected were de novo produced (5.95%) by conceptuses following short-term culture in vitro, but these did display over-representation of secretory activity following pathway analysis. In a separate experiment, we also examined the protein components of EVs isolated from ULF of pregnant and cyclic heifers on Day 16. ULF-derived EVs have been demonstrated to be involved in embryo–maternal communication [21,39,40]; however, the difficulty of identifying conceptus-specific EVs lies in their dual origin as they can either be maternally or conceptus-derived. Surprisingly, no de novo synthesized proteins from the CCM were present in the EVs recovered from ULF. The enriched pathways were mainly related to protein production, including endocytosis. Fructose and mannose metabolism were also highlighted, which could be related to the metabolic demands of the developing conceptus. The GO terms also highlighted endocytosis and protein production, but some other processes related to telomerase activity and regulation of telomeres were also over-represented. 

### 3.1. De novo Synthesised Proteins in Conceptus-Conditioned Media

A relatively low proportion of proteins (5.95% of total proteins identified, Table 1) were identified as containing de novo produced peptides in CCM, i.e., contained heavy labelled amino acids and therefore were produced in vitro. We did identify six different variants of IFNT in the CCM, although only one was heavy labelled in CCM from one conceptus. There was also considerable variation between conceptuses in terms of the proteins produced, and most heavy labelled proteins were de novo produced by one conceptus (277 proteins). Similarly, the proportion of light amino acid labelled proteins present in the CCM are predominantly from one conceptus, illustrating the individual variation between conceptuses. The short duration of culture length (6 h) was used as such filamentous conceptuses do not survive well in vitro. This resulted in a much-reduced exposure time to SILAC media compared to other studies with other types of cultures such as intestinal organoids [30] or bone marrow stromal cells [29]. Similar short incubations have been previously described for BONLAC studies in hippocampal cells (combination of bioorthogonal noncanonical amino acid tagging (BONCAT) and SILAC) [41]. The outcome of these short incubation times will in part depend on the ability of the conceptus to take up those amino acids, and the proportion of light/heavy proteins will also be partially dependent on the protein turnover [42] and their half-life. A longer incubation time using this approach could possibly detect more, but given the limited conceptus survival time under these conditions, this may not be possible.

Three proteins, TKDP1, MERTK, and PARP4, were heavy labelled in the CCM from two or more conceptuses and present in the media from all the conceptuses (Table 3). TKDP1 is produced by the trophoblast with a maximum expression around the beginning of implantation on Day 19 of pregnancy [43] and is likely secreted earlier, e.g., by Day 16 conceptuses in the present study. Other members of the TKFP family (-2, -3, and -4) have previously been identified as expressed in Day 16 conceptuses [44]. MERTK is involved in the regulation of inflammation during pregnancy, restraining toll-like receptor (TLR) signaling [45]. This is a similar signaling pathway that can be activated by a Type I interferon, and conceptus-derived MERTK may play a role in enhancing IFN-mediated signaling in the endometrium. The absence of PARP4, or the downregulation of its coding gene, is related to cancer [46,47,48]. This gene family is related to apoptosis [49] and a potential role for this in Day 16 conceptuses could be regulating cell proliferation. Therefore, the expression of these proteins may contribute to appropriate development of the conceptus and prepare the endometrium for implantation shortly after the pregnancy recognition process.

We did identify a number of other proteins with heavy labelled peptides in CCM from three or more conceptuses, with light labelled versions of these proteins also identified in the CCM from other conceptuses (Table 3) included THO complex subunit 7 homolog, THOC7. Interestingly, THOC7 has been suggested to negatively regulate type I IFN production [50]. Another protein present in the media from five conceptuses (heavy labelled in CCM from three conceptuses) is GPRC5A. This protein belongs to a group of receptors and has been found to be dysregulated in cancer [51] and is a lung cancer suppressor gene [52]. In GPRC5A-knock out mice, prostaglandin E2 synthase (PTGES)/prostaglandin E2 (PGE2) signaling is highly associated with tumorigenesis and metastasis and is related to immune suppression in lung cancer [52]. PGE2 is produced in cattle in response to IFNT to prevent luteolysis and could be related to immune regulation together with GPRC5A. RING finger protein 207 (RNF207) was heavy labelled in five of the CCM and is related to shortened action potential duration in ventricular cardiomyocytes in neonatal rabbits and the knockout of this gene also significantly slows conduction in the ventriculum of the heart of developing zebra fish [53]. Another protein heavy labelled in three CCM was disco interacting protein 2A (DIP2A), which has been previously described as widely expressed in ectoderm-derived tissues in developing embryos and is a potential receptor of FSTL1 and its protective role of cardiomyocytes [54]. This protein, FSTL1, was only detected as light labelled in the CCM from one of the conceptuses in our study. The gene coding for SET domain containing 9 (SETD9), heavy labelled in four CCM and present in media from seven conceptuses, belongs to a greater family, Kmt methyltransferases, that has been found to peak in expression during gastrula stage with a later reduction during embryogenesis in mangrove rivulus fish [55]. Another of the heavy labelled proteins, DExH-box helicase 34 (DHX34), is involved in the complex comprising UPF1 and SMG1, two proteins that are present in the CCM from all the conceptuses as light labelled and that are involved in nonsense-mediated decay [56] and could therefore be important during development although it was only detected in three CCM as heavy labelled. Finally, OTU deubiquitinase 6A (OTUD6A) is a protein of a family of cell signaling cascade regulator. Even though its function is still unknown, it is suggested to be associated with p53 [57]. This last protein was heavy labelled in only three CCM. A low turnover rate and a long half-life of some proteins would explain the low proportion of heavy labelled detected proteins (5.95%) and may further explain the lack of heavy labelled variants of IFNT in the CCM. 

### 3.2. Proteins Detected in the ULF-Derived EVs

This study identified 67 proteins that were exclusively detected in EVs derived from ULF of pregnant heifers. Over-represented GO terms for biological processes were related to different regulatory terms of telomeres, which would be compatible with the morphological and cellular changes that the conceptus is undergoing, i.e., cell division and proliferation. 

Three of these EV pregnancy-specific proteins were common between the EVs of Day 16 pregnant heifers in this study and ULF of Day 16 pregnant heifers from previously published results [25]. These are likely to be exclusive to pregnant heifers, even though they were not detected in ULF of Day 16 pregnant heifers in a different study [19]. From those, SLC1A1 is an amino acid transporter, and clathrin heavy chain, CLTC, is related to the maintenance of the kinetochore fibre tension. Those functions are compatible with the stage of development and high cell division rate of the conceptus. The other protein, transketolase (TKT), is involved in the regulation of excess sugar in the pentose phosphate pathway and has been detected in non EV prepared ULF and CCM in other studies. Differences between studies could be in part due to the approaches used. The study by Forde, McGettigan [25] focused on the proteins present in the ULF of pregnant heifers, whereas in the present one, the EVs were isolated from ULF prior to proteomic analysis by mass spectrometry. These results illustrate the differences between the EV cargo compared to the general ULF composition, probably a consequence of the selective nature of the EV cargo loading, highlighting the potential role of EVs in maternal–embryonic communication. 

### 3.3. Comparison of EVs from ULF vs. CCM Proteins to Determine Those Potentially Involved in IFNT-Independent Communication during the Peri-Implantation Period of Pregnancy in Cattle

When the proteins in the EVs from ULF from both pregnant and cyclic heifers at Day 16 were compared to those light and heavy labelled in the CCM, all groups shared some proteins (Figure 2). However, no de novo produced heavy labelled proteins were present in the EVs from ULF. This indicates that some of the nontraditionally secreted proteins identified in the CCM are potentially mediating communication between the conceptus and the endometrium, but this is likely EV-mediated. Surprisingly, 10 of the 22 proteins only present in cyclic heifers were also present in CCM from all the conceptuses, but not in the EVs from ULF of pregnant heifers. As only the EVs from ULF were analyzed, there is a possibility for a change in the secretion pathway followed during the estrus cycle and pregnancy in which these proteins are not secreted in EVs, but directly to the ULF during pregnancy, either by the conceptus or the endometrium, they would be lost during sample processing.

### 3.4. CCM vs. Previously Published Studies

The proteins present in the CCM from all the conceptuses, as well as those heavy labelled to any extent, were compared to those present in the CCM after 6 and 24 h conceptus culture in a previous study [19]. Only three proteins were common to the four groups. One of them, PAG11, is produced by the trophoblast cells, similar to TKDP1. The other protein, DEAD (Asp-Glu-Ala-Asp) box polypeptide 39 isoform 1 (DDX39A) contributes to genome integrity and telomere protection [58], which protects the dividing somatic cells from a progressive loss of telomeric DNA in successive divisions [59,60]. We have determined that a limited number of proteins are de novo produced by the Day 16 conceptus following short-term culture in vitro.

The most interesting result is the large cohort of 463 proteins that were present in the CCM of all the conceptuses and also after 6 and 24 h of culture in the study by Forde, Bazer [19]. These proteins had GO biological terms related to sensory and chemical perception, telomere regulation and chaperones. The most relevant thing in relation to this group of proteins is that their transcripts were not detected in the endometrium of Day 16 pregnant heifers in a previous study [9]. This indicates that the proteins observed are likely being produced by the conceptuses and, if detected in the ULF, they could be attributable to the conceptus and associated with bilateral communication during pregnancy recognition. Furthermore, they were all light labelled, and therefore produced prior to in vitro culture and not in response to the in vitro environment. Twenty of these proteins were also detected in EVs from ULF of pregnant heifers in the present study (Figure 4), which confirms the EV secretory activity of the conceptus along with the identification of EV markers (HSPA70) among the light labelled proteins in the CCM. Of the 463 proteins in the set, 39 were also detected in EVs from CCM from ovine conceptuses at a similar stage of development, Day 14, after 24 h of in vitro culture [38], eight of which are subunits of the proteasome. Further research will be required to investigate whether these proteins could be potentially conserved between ruminant species and be involved in establishment of pregnancy due to the stage of development at which they have been identified.

In conclusion, we used a SILAC-based approach to investigate the proteins secreted in vitro by Day 16 conceptuses to gain insight into those proteins that are conceptus-derived and may mediate communication during the pregnancy recognition process. We identified a group of 463 proteins in the CCM in the present study and after 6 and 24 h in vitro culture in a previous study [19] that lack transcripts in the endometrium [9] and potentially mediate communication in vivo. Twenty proteins were present only in the EVs from the ULF of Day 16 pregnant heifers and in the CCM from all the conceptuses as well as in previous studies after 6 and 24 h culture [19]. Furthermore, 39 proteins present in CCM in the present and previous studies [19] have been described as present in EVs from CCM of ovine conceptuses at a similar stage of development [38]. We propose that these are involved in molecular communication between the conceptus and endometrium in early pregnancy. 

## 4. Materials and Methods

All experimental procedures involving animals were sanctioned by the Animal Research Ethics Committee of University College Dublin and were licensed by the Health Products Regulatory Authority, Ireland, in accordance with Statutory Instrument No. 543 of 2012 under Directive 2010/63/EU on the Protection of Animals used for Scientific Purposes. Unless otherwise stated all chemicals were sourced from Sigma Aldrich, Dublin, Ireland. 

### 4.1. Experiment 1: Identification of de novo Synthesised Proteins Produced by Day 16 Conceptuses Following Short-Term Culture In Vitro

#### 4.1.1. Animal Synchronization and Sample Collection

The estrous cycles of cross-bred beef heifers were synchronized using a progesterone-releasing intravaginal device (PRID E, 1.55 g progesterone; Ceva Santé Animale, Libourne, France) inserted into the vagina for eight days. On the day of PRID insertion all heifers received a 2 mL intramuscular (i.m.) injection of an analogue of gonadotrophin releasing hormone (Ovarelin; Ceva Santé Animale, Libourne, France, equivalent to 100 μg gonadorelin) and one day prior to PRID E removal all heifers received a 5 mL i.m. injection of a prostaglandin F2α analogue (Enzaprost, Ceva Santé Animal, Libourne, France, equivalent to 25 mg dinoprost). All heifers were monitored for standing estrus and were artificially inseminated (*n* = 10) twice, 12 h apart, with semen from a bull of proven fertility. All heifers were slaughtered on Day 16 of the estrous cycle/ early pregnancy (estrus = Day 1) at a commercial abattoir. After slaughter, the reproductive tracts were recovered and transported at room temperature for approximately 40 min to the laboratory where the uterine horn ipsilateral to the CL was noted and both uterine horns were flushed separately with 10 mL of PBS (pH 7.2). The volume of recovered ULF was noted and only those with an appropriately elongated conceptus were further processed. Conceptuses (*n* = 8) were individually placed in a six-well dish with 10 mL arginine- and lysine-depleted culture medium (SILAC DMEM, Thermo Fisher, UK) supplemented with heavy labelled arginine (L-arginine 13C6, Thermo Fisher, UK) and lysine (L-lysine 13C6, Thermo Fisher, UK) and were cultured for 6 h at 38.5 °C, 5% CO_2_ and 5% O_2_. At one-hour intervals 500 μL of conceptus conditioned medium (CCM) was removed and after 6 h of culture, media was clarified by centrifugation at 3000× *g* for 15 min at 4 °C. The supernatant was recovered into a new RNase DNase free tube and snap frozen in liquid nitrogen and stored at −80 °C prior to processing. Contemporaneous blanks (medium without a conceptus) were also cultured in parallel and processed as above. Only the 6 h time-point recovered media was used for the present study.

#### 4.1.2. Analysis of Protein Content of Conceptus-Conditioned Medium (CCM)

Samples from the 6 h CCM time-point were concentrated to approximately 50 µl using Microcon centrifugal filter units with a 3 kDa cut-off (Merck Millipore, Burlington, MA, USA). Twenty-five μL of 3× laemmli buffer was then added and the samples boiled for 15 min prior to loading onto a 10% SDS-polyacrylamide gel. The gel was resolved at 125 V until the dye front had entered the separating gel and then at 200 V until the dye front had moved 3 cm into the separating gel. The gel was briefly stained in 0.1% Coomassie brilliant blue R in 50% methanol and then destained in 50% methanol; 7% acetic acid. To ensure complete removal of Coomassie and to remove SDS, the gel slices were then subjected to four cycles of the following destaining process: Incubation in 300 μL 50 mM ammonium bicarbonate for 10 min followed by the addition of 600 μL acetonitrile and incubation for a further 45 min. After the final destain step, the gel slices were incubated in water for 30 min, cut into 1 mm square pieces and subjected to in-gel tryptic digestion using a DigestPro automated digestion unit (Intavis, Cologne, Germany). The resulting peptides were fractionated using an Ultimate 3000 nano-LC system in line with an Orbitrap Fusion Tribrid mass spectrometer (Thermo Scientific, Waltham, MA, USA). In brief, peptides in 1% (vol/vol) formic acid were injected onto an Acclaim PepMap C18 nano-trap column (Thermo Scientific, Waltham, MA, USA). After washing with 0.5% (vol/vol) acetonitrile 0.1% (vol/vol) formic acid, peptides were resolved on a 250 mm × 75 μm Acclaim PepMap C18 reverse phase analytical column (Thermo Scientific, Waltham, MA, USA) over a 150 min organic gradient, using seven gradient segments (1%–6% solvent B over 1 min, 6%–15% B over 58 min, 15%–32% B over 58 min, 32%–40% B over 5 min, 40%–90% B over 1 min, held at 90% B for 6 min, and then reduced to 1% B over 1 min) with a flow rate of 300 NL/min. Solvent A was 0.1% formic acid and Solvent B was 80% aqueous acetonitrile in 0.1% formic acid. Peptides were ionized by nanoelectrospray ionization at 2.2 kV using a stainless-steel emitter with an internal diameter of 30 μm (Thermo Scientific, Waltham, MA, USA) and a capillary temperature of 250 °C. 

All spectra were acquired using an Orbitrap Fusion Tribrid mass spectrometer controlled by Xcalibur 3.0 software (Thermo Scientific, Waltham, MA, USA) and operated in data-dependent acquisition mode. FTMS1 spectra were collected at a resolution of 120,000 over a scan range (m/z) of 350–1550, with an automatic gain control (AGC) target of 400,000 and a max injection time of 100 ms. Precursors were filtered according to charge state (to include charge states 2–7), with monoisotopic peak determination set to peptide and using an intensity range from 5 × 10^3^ to 1 × 10^20^. Previously interrogated precursors were excluded using a dynamic window (40 s ± 10 ppm). The MS2 precursors were isolated with a quadrupole mass filter set to a width of 1.6 m/z. ITMS2 spectra were collected with an AGC target of 5000, max injection time of 50 ms and HCD collision energy of 35%.

#### 4.1.3. Data Analysis

The raw proteomic mass spectrometry data files were processed using Proteome Discoverer software v1.4 (Thermo Scientific, Waltham, MA, USA) and searched against the UniProt *Bos taurus* database (downloaded March 2019; 32,159 entries) using the SEQUEST algorithm. Peptide precursor mass tolerance was set at 10 ppm, and MS/MS tolerance was set at 0.6 Da. Search criteria included carbamidomethylation of cysteine (+57.0214) as a fixed modification and oxidation of methionine (+15.9949) and appropriate SILAC labels (^13^C_6_-Arg and ^13^C_6_-Lys) as variable modifications. Searches were performed with full tryptic digestion and a maximum of two missed cleavage events were allowed. The reverse database search option was enabled and all peptide data was filtered to satisfy false discovery rate (FDR) of 5%. 

Proteins detected in any of the three media-only samples (contemporaneous blanks) had their Uniprot IDs combined with “cat | sort -u”, and then removed from each individual conceptus protein list with “diff –y | grep –P ‘[>\|]’” Unix/Bash commands. A Venn Diagram-like textual table was generated using the BASH pipeline to allow a searchable data base containing shared or specific proteins from *n* = 8 CCM data sets, as well as retrieve both “Scores” and “Heavy/Light Variability” metrics from any selected list of target proteins. The proteins detected in CCM were submitted for pathway analysis on DAVID (DAVID Bioinformatics Resources 6.8, NIAD, https://david.ncifcrf.gov/) [33,34]. Three different analyses were performed. The first analysis included all the proteins present in the CCM from all eight conceptuses, independent of the presence of peptide fragments containing heavy labelled amino acids. A second analysis included all proteins with a peptide fragment containing a heavy label in CCM from at least two different conceptuses. Finally, the last analysis included all proteins that were heavy labelled in the media from at least one conceptus but present in the media from all conceptuses (i.e., heavy labelled in the media from one conceptus and light labelled in the media from seven conceptuses). The same protein groups were also analyzed for GO terms with the GO enrichment analysis tool (http://geneontology.org/) [35,36,37]. Comparisons between studies and groups and Venn diagrams were performed with the tool Venny (version 2.1, CNB-CSIC, Madrid, Spain; https://bioinfogp.cnb.csic.es/tools/venny/) [61].

### 4.2. Experiment 2: Proteomic Component of Extracellular Vesicles Obtained from the Uterine Luminal Fluid of Cyclic and Pregnant Heifers on Day 16

#### 4.2.1. Animal Synchronization and Sample Collection

The estrous cycles of cross-bred beef heifers were synchronized as described above. Heifers were monitored for standing estrus and randomly assigned to either a cyclic control (*n* = 7) or an artificially inseminated group (*n* = 11). All heifers were slaughtered on Day 16 of the estrous cycle/early pregnancy (estrus = Day 0) at a commercial abattoir. After slaughter the reproductive tracts were recovered and kept on ice prior to processing for sample collection at the abattoir. Uterine horns ipsilateral and contralateral to the CL were flushed separately with 10 mL of PBS (pH 7.2) as described above. The volume of recovered fluid was noted and, in the inseminated group, only those with an appropriately elongated conceptus were further processed. The recovered fluid was clarified by centrifugation at 3000× *g* for 15 min at 4 °C. The supernatant was recovered into a new RNase DNase free tube, gently inverted 10 times to ensure homogeneity, aliquoted into 1 mL aliquots and snap frozen in liquid nitrogen and stored at −80 °C prior to processing for extracellular vesicle (EV) analyses. 

#### 4.2.2. Extraction of Extracellular Vesicles from Uterine Luminal Fluid and Proteomic Analysis

The EV isolation protocol was as described by Burns, Brooks [21]. The ULF was clarified and filtered with a 0.22 μm PVDF syringe filter (EMD Millipore, Billerica, MA, USA) to remove large microvesicles. EVs were isolated from by the addition of 200 µL ExoQuick-TC (System Biosciences, Palo Alto, CA, USA) precipitation solution per mL of filtered ULF. Tubes were incubated overnight at 4 °C and then centrifuged at 15,000× *g* for 30 min at 4 °C to recover EVs. Pellets were suspended in sterile PBS and evaluated using a NanoSight LM-10 instrument (NanoSight, Amesbury, UK) calibrated with 100 nm polystyrene beads (Polysciences, Warrington, PA, USA). Videos were captured (camera level 13–14, syringe pump speed 30, temperature control setting 25 °C) using the standard measurement protocol of five 60 s videos followed by processing with NTA software (NanoSight) to track each visible particle. The Stokes–Einstein equation was employed by the software to determine the size distribution and number of particles (concentration) within each sample. The protein content of these EVs was analysed by mass spectrometry by the Charles W. Gehrke Proteomics Center at the University of Missouri as previously described [5]. 

## Figures and Tables

**Table 1:** Number and percentage of the proteins present in CCM identified via nano-LC MS/MS proteomics of SILAC-labelled media minus Arginine and Lysine supplemented with heavy forms of these amino acids (*n* = 8 CCM: *n* = 3 contemporaneous blanks) from Day 16 in vivo produced conceptuses after 6 h culture.

Number of Conceptuses	Proteins	% of Total	Heavy Labelled Proteins	% Heavy Labelled Proteins	% Heavy/Total Proteins
8	1576	14.17	0	0	0
≥7	2180	19.60	0	0	0
≥6	2757	24.79	0	0	0
≥5	3314	29.80	2	0.3	0.01
≥4	3997	35.94	3	0.45	0.02
≥3	4939	44.41	9	1.36	0.08
≥2	6680	60.06	51	7.7	0.46
≥1 (total)	11,122	100	662	100	5.49

**Table 2:** Top 10 overrepresented pathways from 1576 proteins identified in the in conceptus-conditioned media (CCM) (*n* = 8) from all the conceptuses via nano-LC MS/MS proteomic analysis of SILAC-labelled media (minus Arginine and Lysine supplemented with heavy forms of these amino acids: *n* = 8 CCM, *n* = 3 contemporaneous blanks) from Day 16 in vivo produced conceptuses after 6 h culture.

GO Terms	Count	%	*p* Value	List Total	Pop Hits	Pop Total	Fold Enrichment
Proteasome	31	2.032787	3.80 × 10^−17^	926	46	7550	5.494647
Citrate cycle (TCA cycle)	17	1.114754	6.21 × 10^−8^	926	30	7550	4.62023
Pyruvate metabolism	21	1.377049	1.83 × 10^−9^	926	38	7550	4.505797
Other glycan degradation	11	0.721311	4.21 × 10^−5^	926	20	7550	4.484341
Propanoate metabolism	14	0.918033	2.89 × 10^−6^	926	26	7550	4.390264
Carbon metabolism	55	3.606557	7.77 × 10^−22^	926	109	7550	4.114075
Amino sugar and nucleotide sugar metabolism	24	1.57377	1.23 × 10^−9^	926	48	7550	4.076674
2-Oxocarboxylic acid metabolism	9	0.590164	6.95 × 10^−4^	926	18	7550	4.076674
Sulfur metabolism	5	0.327869	0.025586	926	10	7550	4.076674
Glyoxylate and dicarboxylate metabolism	12	0.786885	1.18 × 10^−4^	926	26	7550	3.763084

**Table 3:** Proteins present in CCM identified via nano-LC MS/MS proteomics of SILAC-labelled media minus Arginine and Lysine supplemented with heavy forms of these amino acids (*n* = 8 CCM: *n* = 3 contemporaneous blanks) from Day 16 in vivo produced conceptuses after 6 h culture with heavy isotopes (H) in three or more CCM (B–J), and their presence as light (L) isotopes or absence (“0”). The total of CCM presenting heavy (Count H labelled) or light isotopes (Count L labelled) is added up for each protein under Total count. The Protein ID corresponds to uniport.org.

Protein	Protein Name	Protein ID	B	C	D	E	F	H	I	J	Count H Labelled	Count L Labelled	Total Count
THO complex subunit 7 homolog	THOC7	F1MSQ1	H	H	L	H	0	0	H	H	5	1	6
RING finger protein 207	RNF207	A0JNG4	H	H	H	0	H	0	H	0	5	0	5
SET domain containing 9	SETD9	F1MWB0	H	H	H	L	L	H	L	0	4	3	7
MER proto-oncogene, tyrosine kinase	MERTK	F1N381	H	L	H	L	L	H	L	L	3	5	8
G protein-coupled receptor class C group 5 member A	GPRC5A	F1N6N2	H	H	0	L	L	H	0	0	3	2	5
DExH-box helicase 34	DHX34	E1BJ90	0	H	H	0	0	0	H	0	3	0	3
Disco interacting protein 2 homolog A	DIP2A	F1MPW1	0	0	0	H	0	0	H	H	3	0	3
Uncharacterized protein		G3X6J6	H	0	0	H	0	0	H	0	3	0	3
OTU deubiquitinase 6A	OTUD6A	G5E596	H	0	H	0	0	0	H	0	3	0	3

**Table 4:** Over-represented pathways in the subset of heavy labelled proteins present in the CCM from two or more conceptuses via nano-LC MSMS proteomics of SILAC-labelled media minus Arginine and Lysine supplemented with heavy forms of these amino acids (*n* = 8 CCM, *n* = 3 contemporaneous blanks) from Day 16 in vivo produced conceptuses after 6 h culture.

KEGG Pathway	Count	%	*p* Value	List Total	Pop Hits	Pop Total	Fold Enrichment
Bile secretion	3	6.818182	0.006783	15	68	7550	22.20588
Aldosterone synthesis and secretion	3	6.818182	0.009068	15	79	7550	19.11392
Salivary secretion	3	6.818182	0.009973	15	83	7550	18.19277
Ovarian steroidogenesis	2	4.545455	0.088901	15	50	7550	20.13333

**Table 5:** Concentration, mean and mode of the Extracellular Vesicles from ULF of Day 16 pregnant and cyclic heifers via NanoSight analysis.

Group	Concentration (Particles/mL)	Mean Diameter (nm)	Mode Diameter (nm)
Pregnant Day 16 Heifer 1	1.25 × 10^12^	128.7	99.1
Pregnant Day 16 Heifer 2	3.16 × 10^11^	98.1	95.4
Pregnant Day 16 Heifer 3	1.04 × 10^12^	127.4	92.4
Pregnant Day 16 Heifer 4	6.01 × 10^11^	111.2	91.6
Pregnant Day 16 Heifer 5	1.91 × 10^12^	138.5	106.7
Pregnant Day 16 Heifer 6	1.32 × 10^12^	138.8	106.7
Pregnant Day 16 Heifer 7	1.63 × 10^12^	141.5	100.0
Cyclic Day 16 Heifer 1	2.86 × 10^11^	115.2	92.1
Cyclic Day 16 Heifer 2	1.34 × 10^12^	146.7	103.3
Cyclic Day 16 Heifer 3	2.04 × 10^12^	151.1	108.8
Cyclic Day 16 Heifer 4	9.01 × 10^11^	123.4	100.0
Cyclic Day 16 Heifer 5	1.05 × 10^12^	162.0	113.3
Cyclic Day 16 Heifer 6	1.05 × 10^12^	111.4	101.8

**Table 6:** Eleven over-represented pathways of 67 proteins only present in the EVs from ULF in Day 16 pregnant heifers (*n* = 6) compared to cyclic heifers (*n* = 5) via nano-LC MS/MS proteomics.

KEGG Pathway	Count	%	*p* Value	List Total	Pop Hits	Pop Total	Fold Enrichment
Collecting duct acid secretion	3	5.172414	7.59 × 10^−3^	38	27	7550	22.07602
Synaptic vesicle cycle	6	10.34483	1.22 × 10^−5^	38	63	7550	18.92231
Endocrine and other factor-regulated calcium reabsorption	3	5.172414	1.78 × 10^−2^	38	42	7550	14.19173
Tight junction	3	5.172414	0.067422	38	87	7550	6.85118
Rheumatoid arthritis	3	5.172414	0.078554	38	95	7550	6.274238
Systemic lupus erythematosus	5	8.62069	1.08 × 10^−2^	38	178	7550	5.581017
Carbon metabolism	3	5.172414	0.099294	38	109	7550	5.468373
Phagosome	4	6.896552	4.16 × 10^−2^	38	158	7550	5.02998
Endocytosis	6	10.34483	6.23 × 10^−3^	38	243	7550	4.905783
Protein processing in endoplasmic reticulum	4	6.896552	0.049135	38	169	7550	4.702585
Biosynthesis of antibiotics	4	6.896552	0.079032	38	206	7550	3.857946

**Table 7:** Top 10 of 30 over-represented pathways of the 463 proteins present in the CCM from all the conceptuses via nano-LC MSMS proteomics of SILAC-labelled media minus Arginine and Lysine supplemented with heavy forms of these amino acids (*n* = 8 CCM, *n* = 3 contemporaneous blanks) from Day 16 in vivo produced conceptuses after 6 h culture, and previously reported proteins present in CCM from Day 16 in vivo produced conceptuses after 6 and 24 h culture [19].

KEGG Pathway	Count	%	*p* Value	List Total	Pop Hits	Pop Total	Fold Enrichment
Glyoxylate and dicarboxylate metabolism	9	1.978022	6.96 × 10^−6^	317	26	7550	8.244358
Proteasome	15	3.296703	2.71 × 10^−9^	317	46	7550	7.766424
Other glycan degradation	6	1.318681	0.001147	317	20	7550	7.14511
Pentose phosphate pathway	8	1.758242	9.06 × 10^−5^	317	27	7550	7.056899
2-Oxocarboxylic acid metabolism	5	1.098901	0.005782	317	18	7550	6.615843
Ribosome	36	7.912088	4.09 × 10^−19^	317	134	7550	6.398606
Biosynthesis of amino acids	19	4.175824	3.97 × 10^−10^	317	71	7550	6.373573
Citrate cycle (TCA cycle)	8	1.758242	1.86 × 10^−4^	317	30	7550	6.351209
Cysteine and methionine metabolism	10	2.197802	1.96 × 10^−5^	317	38	7550	6.267641
Glycosaminoglycan degradation	6	1.318681	0.002246	317	23	7550	6.213139

## Supplementary Materials

Supplementary materials can be found at https://www.mdpi.com/1422-0067/21/8/2870/s1.
